# Diet quality is associated with adipose tissue and muscle mass: the Coronary Artery Risk Development in Young Adults (CARDIA) study

**DOI:** 10.1002/jcsm.13399

**Published:** 2023-12-12

**Authors:** Masoud Isanejad, Lyn M. Steffen, James G. Terry, James M. Shikany, Xia Zhou, So‐YunYi, David R. Jacobs, John Jeffrey Carr, Brian T. Steffen

**Affiliations:** ^1^ Institute of Life Course and Medical Sciences University of Liverpool Liverpool UK; ^2^ Division of Epidemiology and Community Health, School of Public Health University of Minnesota Minneapolis Minnesota USA; ^3^ Department of Radiology Vanderbilt University Medical Center Nashville Tennessee USA; ^4^ Division of Preventive Medicine, Heersink School of Medicine University of Alabama at Birmingham Birmingham Alabama USA; ^5^ Division of Computational Health Science, Department of Surgery, School of Medicine University of Minnesota Minneapolis Minnesota USA

**Keywords:** Diet quality, Intramuscular adipose tissue, Muscle mass, Muscle quality, Visceral adipose tissue

## Abstract

**Background:**

Aging is associated with changes in body composition, and preventing loss of muscle mass and accumulation of excess adipose tissue in middle‐aged adults may reduce age‐related conditions at older ages. Dietary intake is one lifestyle factor shown to improve or maintain body composition. However, few studies have examined the Healthy Eating Index2015 (HEI2015), a measure of diet quality, and the association with body composition in adult men and women.

**Methods:**

Participant data (*n* = 3017) from the Coronary Artery Risk Development in Young Adults (CARDIA) study were used to examine the associations of the HEI2015 with body composition measures at Year 25 (Y25), including (1) 25 year‐change in weight, body mass index (BMI), and waist circumference and (2) a computed tomography (CT) scan at Y25 measured muscle mass, muscle quality (better quality = less lipid within the muscle), and adipose tissue depots visceral adipose tissue (VAT), subcutaneous adipose tissue (SAT), and adipose within skeletal muscle (intermuscular adipose tissue; IMAT). Dietary intake was assessed by a diet history three times over 20 years, at years 0, 7, and 20. HEI2015, averaged over three exams, was created and categorized into quintiles. Multiple regression analysis evaluated the associations of body composition stratified across quintiles of HEI2015 adjusted for demographic characteristics, energy intake, lifestyle factors, and baseline anthropometric measures as appropriate. Race–sex interaction was tested (*P*_interaction_ > 0.30).

**Results:**

Over 25 years of follow‐up, averaged HEI2015 was significantly and inversely associated with weight gain (Quintile 1 (Q1) 37.3 lb vs. 32.9 in Q5; *P*_trend_ = 0.01), change in BMI (Q1 5.8 kg/m^2^ vs. 5.0 in Q5; *P*_trend_ = 0.005), and change in waist circumference (Q1 17.5 cm vs. 15.2 cm in Q5; *P*_trend_ < 0.001). By Y25, HEI2015 was inversely associated with VAT Q1 136.8 cm^3^ vs. 116.6 in Q5; *P*_trend_ < 0.001) and IMAT volumes (Q1 9.52 vs. 8.12 cm^3^ in Q5; *P*_trend_ < 0.001). Although total muscle volume declined (*P*_trend_ = 0.03), lean muscle mass volume was similar across quintiles (*P*_trend_ = 0.55). The IMAT/total muscle mass ratio declined across HEI2015 quintiles (*P*_trend_ < 0.001). Finally, higher HEI2015 was associated with better muscle quality at Y25 (higher value = less lipid within the muscle; Q1 41.1 vs. 42.2 HU in Q5; *P*_trend_ = 0.002). HEI2015 was nonlinearly, but inversely, associated with SAT (nonlinear *P* = 0.011).

**Conclusions:**

Improving diet quality in young to middle‐aged adults is a recommended strategy to promote better measures of body composition. Our study findings suggest that healthier food choices may influence body composition.

## Introduction

Aging is associated with potentially unfavourable changes in body composition, including loss of lean muscle mass, lower muscle quality (i.e., as indicated by higher muscle fat infiltration) and accumulation of organ‐related fat mass, such as visceral adipose tissue (VAT) and adipose between skeletal muscle bundles (intermuscular adipose tissue; IMAT), and of subcutaneous adipose tissue (SAT).^1^, ^2^ Preventing loss of muscle mass and accumulation of excess adipose tissue in middle‐aged adults may reduce age‐related conditions, such as sarcopenia, sarcopenic obesity, and complications from sarcopenia, at older ages.^3^, ^4^ Therefore, it is important to identify strategies to increase or maintain muscle mass and reduce the accumulation of adipose tissue prior to old age.^5^

In addition to physical activity, it is well established that high protein intake promotes and maintains muscle mass^6^ and at the same time, protein intake potentially reduces the accumulation of fat mass.^7^ In addition to protein‐rich foods, there is growing interest in the benefits of a healthy diet pattern on muscle mass and adipose tissue.^8^ In Australian men and women, a traditional diet pattern—high in animal protein, vegetables and whole grains—was associated with greater skeletal muscle mass index as measured by dual‐energy X‐ray absorptiometry (DXA).^9^, ^10^ In the Korea National Health and Examination Survey, middle‐aged and elderly adults who consumed a diet pattern high in white rice, fish, and seaweeds were less likely to have low DXA‐measured skeletal muscle mass index compared with those consuming a diet pattern high in condiments, vegetables and meats.^11^ In US adults, higher diet quality was inversely associated with VAT in young, middle‐aged and older multiethnic adults.^12^, ^13^ Higher quality diets typically include recommended amounts of protein food sources (meat, poultry, fish, eggs, dairy products, nuts, and/or meat alternatives), whole grain products, fruit, and vegetables, and lower intakes of refined grain products, added sugar, saturated fat and sodium.

Yet, robust studies examining associations of US Dietary Guidelines for Americans (DGA) Healthy Eating Index 2015 (HEI2015) with muscle mass, muscle quality, and adipose tissue depots in middle‐aged adults are lacking. And while dietary intake has been associated with weight, height, waist circumference, and DXA‐measured skeletal muscle mass index,^9^, ^10^, ^11^ computed tomography (CT) scans provide more precise measurements of muscle mass, muscle quality and regional adipose tissue volumes. Therefore, the aim of this study was to assess the associations of the HEI2015 with body composition measures, including anthropometric measures and CT scan‐measured muscle mass, muscle quality, and adipose tissue depots VAT, SAT and IMAT in women and men enrolled in the Coronary Artery Risk Development in Young Adults (CARDIA) study. Because muscle mass is typically greater in men than women,^14^ the role of sex as a modifying factor was examined.

## Subjects and methods

### Study population

**Figure 1:**
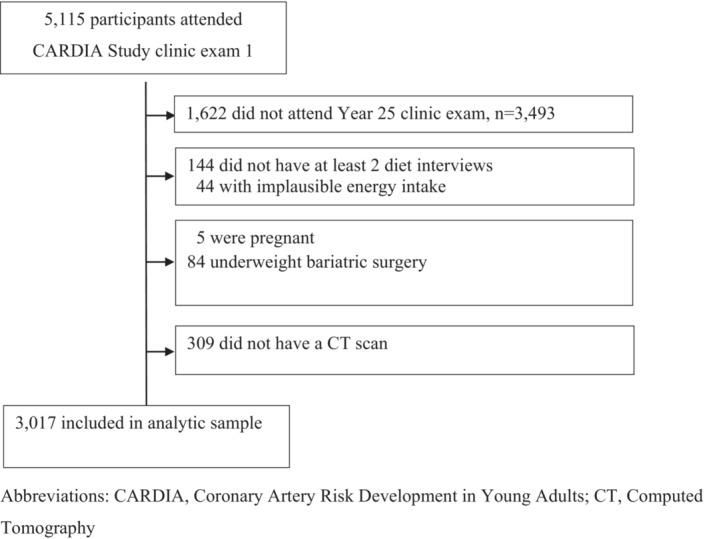
Flowchart of exclusion criteria (exclusions are not mutually exclusive).

The CARDIA study enrolled 5115 participants aged 18 to 30 years between 1985 and 1986 at field centres located in Birmingham, AL; Chicago, IL; Minneapolis, MN; and Oakland, CA. The current prospective study includes data from participants who reported dietary intake at year 0 (Y0, baseline) and year 7 (Y7) or year 20 (Y20); and those who underwent CT scan imaging at the Y25 CARDIA clinic examination (*n* = 3189 of *n* = 3499 year 25 participants; 91%). Specifically, exclusions include those who did not attend Y25 exam (*n* = 1616), did not have at least 2 diet interviews (*n* = 144); had implausible energy intake [<600 and >6000 kcal/day for women (*n* = 20) and <800 and >8000 kcal/day for men (*n* = 24)]; were pregnant (*n* = 5), underwent bariatric surgery before the Y25 CT scan (*n* = 84), or did not have a CT scan (*n* = 309). The sample size in these analyses was *n* = 3017, including 1687 women and 1330 men. A flowchart of participant exclusion criteria in these analyses is shown in Figure 1.

### Dietary assessment

Dietary intake was assessed by the interviewer‐administered CARDIA Diet History^15^ at Y0, Y7 and Y20 that provided quantitative information about usual food and beverage intakes during the past month. Trained and certified interviewers asked 100 open‐ended questions, including brand name and food preparation, if known, about food and beverages consumed daily, weekly or monthly. Food models were used to assist the participant in estimating portion size. Foods were assigned according to the food grouping system in the Nutrition Data System for Research (NDSR) developed at the University of Minnesota Nutrition Coordinating Center. NDSR output includes daily nutrient intake and food and beverage group intake (servings per day). Food and beverage groups include fruit, fruit juice, vegetables, whole grains, refined grains, legumes, nuts, dairy products, fish and seafood, poultry, red and processed meat, candy, sugar sweetened beverages, diet beverages, coffee and tea.

### The healthy eating index: HEI2015

The HEI2015, a higher score representing better diet quality, is based on the 2015–2020 DGAs.^16^ As an additional component, ‘added sugar’ was incorporated into the HEI in 2015, resulting in a total of 13 dietary components. Each of the 13 HEI2015 components is scored on a density basis per 1000 kcal, with the exception of fatty acids, which is a ratio of unsaturated to saturated fatty acids, and added sugar and saturated fat that are represented as % of energy.

### Other measurements

Standard questionnaires were used to obtain self‐reported demographic and behavioural information. Age, sex, race, education, and cigarette smoking status were ascertained by self‐administered questionnaires at each examination. Educational status was categorized as greater than high school (yes/no). Self‐reported current smoking status and current alcohol consumption status were classified as yes/no. Height and weight of participants were measured at each examination and recorded to the nearest 0.5 cm and 0.2 kg, respectively. Body mass index (BMI) was defined as weight (in kg) divided by height squared (m^2^). A physical activity score was derived from the CARDIA Physical Activity Questionnaire, which is a simplified version of the Minnesota Leisure Time Physical Activity Questionnaire,^17^ at each examination.

### CT scan measures of muscle mass and adipose tissue

CT scans of the abdominal adipose tissue and abdominal muscle composition at Y25 were performed using 64 channel multi‐detector CT scanners [GE 750HD and GE Light‐Speed VCT (GE Healthcare, Waukesha, WI) at the Birmingham and Oakland Centers, respectively; Siemens Sensation (Siemens Medical Solutions, Erlangen, Germany) at the Chicago and Minneapolis Centers] with previously detailed standardized multi‐centre CT protocols for acquisition and quality assurance.^18^ In the abdomen, axial thin slice images of 0.6–0.625 mm thickness, as well as reconstructions in 1.2–1.25 mm and standard 2.5–3 mm thicknesses, were acquired along with frontal and lateral scouts. CT images were electronically transmitted using secure protocol to the central CT reading centre at Wake Forest University Medical Center, Winston‐Salem, NC.

The National Institutes of Health's Center of Information Technology Medical Image Processing, Analysis, and Visualization (MIPAV) application https://mipav.cit.nih.gov/to was used to perform quantitative measurements. Abdominal adipose tissue volumes (SAT and VAT, cm^3^) along with muscle composition [fat, lean, and total muscle mass volumes (cm^3^) and attenuation (HU)] were quantified using a customized MIPAV plug‐in developed by study investigators.^19^ Muscle attenuation has been suggested as a marker of muscle quality (less lipid within the muscle).^20^ The left‐ and right‐side measures for each muscle group were highly correlated, so mean lean, adipose, and total volumes and mean attenuations of the left and right sides were calculated and analysed for each muscle group separately, and overall for all abdominal muscles. Overall (intra‐ and inter‐reader) technical error in re‐analysis of 156 pairs of scans was 7.7% for psoas muscle total volume with correlations for rereads >0.95.^19^ The interclass correlation coefficient for inter‐reader comparisons was 0.98 for VAT, and intra‐ and inter‐reader error were 2.4% and 6.7%, respectively, in 156 scans that were blinded and reevaluated.

### Statistical methods

SAS, version 9.4 (SAS Institute Inc.; Cary, NC) was used to analyse the data. HEI2015 scores were created for each of Y0, Y7 and Y20 diet data. The within‐person correlation coefficients between Y0, Y7 and Y20 HEI2015 scores ranged from 0.50–0.53 (*P* < 0.001). This strong tracking suggests that the HEI score at 1 year carries information about the HEI score at each consecutive year. Therefore, it seemed prudent to average previous dietary intake (reflecting many years of dietary habits and change in the food supply) to predict body composition measures. Further, the HEI2015 scores were averaged to improve the precision to potentially strengthen the diet‐body composition associations.^21^ Quintiles were created after HEI2015 scores were averaged. Baseline characteristics were presented as means and standard errors (SE) or frequencies (SE). Covariates energy intake and physical activity were also averaged over Y0, Y7 and Y20.^21^ General linear regression evaluated the associations of demographic characteristics, nutrient and food intakes, muscle mass, muscle quality, adipose tissue depots (VAT, SAT and IMAT) and anthropometric measures stratified across quintiles of HEI2015. The models assessing baseline demographic characteristics and dietary intake were adjusted for age, sex, race, field centre, education and energy intake (Tables 1 and 2). Models assessing body composition measures were adjusted for age, sex, race, field centre, education, height, current smoking status, current drinking status and averaged energy intake and physical activity. In addition, the models evaluating change in BMI, weight, and waist circumference were also adjusted for the respective baseline measure. In another model, we assessed mediation of hormone replacement therapy use and prevalent hypertension, high cholesterol diabetes, and cardiovascular disease on the diet‐body composition associations. Effect modification by sex was tested on the associations of HEI2015 with muscle mass and adipose tissue depots. However, the interaction terms were not statistically significant (*P*_interaction_ ranged from 0.34–0.79).

**Table 1:** Baseline (1985–86) characteristics stratified across quintiles of HEI2015 diet quality core among CARDIA participants, *n* = 3017

	Baseline quintiles of HEI2015 diet quality score					
Baseline characteristics*	1 (*n* = 603)	2 (*n* = 604)	3 (*n* = 603)	4 (*n* = 604)	5 (*n* = 603)	*P*_trend_
HEI2015, mean (range)	40.1 (24.5 < 45.5)	48.7 (45.5 < 51.6)	54.8 (51.6 < 57.9)	61.2 (57.9 < 65.3)	72.2 (>65.3)	
Age	24.4 (0.15)	24.7 (0.14)	25.2 (0.14)	25.4 (0.14)	25.8 (0.15)	<0.001
Sex, women %	49.8 (1.88)	53.2 (1.85)	52.7 (1.82)	57.8 (1.83)	66.2 (1.91)	<0.001
Race, White %	45.3 (1.94)	43.9 (1.89)	51.4 (1.88)	55.5 (1.88)	69.9 (1.96)	<0.001
Education, >HSl	42.7 (1.84)	63.3 (1.81)	62.7 (1.79)	71.1 (1.79)	76.7 (1.87)	<0.001
Current smoking, %	33.3 (1.78)	28.2 (1.75)	26.0 (1.73)	24.3 (1.73)	19.4 (1.82)	<0.001
Current alcohol, %	85.14 (1.40)	88.08 (1.37)	87.43 (1.35)	88.74 (1.36)	84.05 (1.43)	0.74
Physical activity score	328.8 (11.2)	375.5 (10.9)	417.4 (10.9)	454.5 (10.9)	507.9 (11.4)	<0.001
BMI, kg/m^2^	24.0 (0.19)	24.6 (0.19)	24.8 (0.19)	24.4 (0.19)	24.1 (0.20)	0.94
Height, cm	169.7 (0.28)	170.2 (0.27)	170.2 (0.27)	170.7 (0.27)	170.7 (0.29)	0.09
Weight, lb	152.6 (1.32)	157.2 (1.28)	158.8 (1.27)	156.6 (1.28)	155.2 (1.34)	0.31
Waist circ, cm	77.0 (0.42)	78.3 (0.41)	78.5 (0.41)	77.3 (0.41)	76.2 (0.43)	0.09

Abbreviations: BMI, body mass index; HEI2015, Healthy Eating Index 2015; HS, high school; waist circ, waist circumference.

* Adjusted for age, sex, race, field centre, education and energy intake.

**Table 2:** Baseline (1985–86) dietary intake stratified across quintiles of HEI2015 diet quality score among CARDIA participants, *n* = 3017

	Baseline quintiles of HEI2015 diet quality score					
Dietary intake*	1 (*n* = 603)	2 (*n* = 604)	3 (*n* = 603)	4 (*n* = 604)	5 (*n* = 603)	
HEI2015 mean (range)	40.1 (24.5 < 45.5)	48.7 (45.5 < 51.6)	54.8 (51.6 < 57.9)	61.2 (57.9 < 65.3)	72.2 (>65.3)	
***Nutrients***						*P*_trend_
Energy, kcal	2890 (51.3)	2999 (50.0)	2798 (49.6)	2708 (49.7)	2619 (52.1)	<0.001
Total fat, g	92.0 (0.95)	94.9 (0.93)	95.5 (0.92)	93.6 (0.93)	91.9 (0.97)	0.63
SFA, g	35.0 (0.40)	35.2 (0.39)	34.8 (0.38)	33.2 (0.38)	30.1 (0.40)	<0.001
MUFA, g	33.6 (0.42)	34.6 (0.41)	35.0 (0.41)	34.4 (0.41)	33.9 (0.43)	0.79
PUFA, g	16.7 (0.31)	18.0 (0.31)	18.5 (0.30)	19.2 (0.31)	20.9 (0.32)	<0.001
n3 fatty acids, g	1.64 (0.05)	1.84 (0.05)	2.05 (0.05)	2.12 (0.05)	2.32 (0.05)	<0.001
Carbohydrate, g	199.5 (3.55)	216.3 (3.47)	224.2 (3.43)	216.0 (3.44)	204.6 (3.61)	0.40
Protein, g	60.9 (0.80)	71.2 (0.78)	75.1 (0.77)	76.9 (0.78)	83.3 (0.81)	<0.001
Fibre, g	11.3 (0.27)	13.4 (0.26)	16.2 (0.26)	19.0 (0.26)	25.9 (0.27)	<0.001
Added sugar, g	104.7 (1.81)	83.3 (1.76)	72.8 (1.74)	70.5 (1.75)	61.6 (1.84)	<0.001
***Food intake (sv/day)***						
Dairy	2.6 (0.09)	3.3 (0.09)	3.3 (0.09)	3.3 (0.09)	3.1 (0.09)	0.001
Fruit, fruit juice	0.8 (0.08)	1.4 (0.08)	2.1 (0.08)	2.7 (0.08)	3.7 (0.08)	<0.001
Fruit w/o juice	0.5 (0.06)	0.9 (0.05)	1.3 (0.05)	1.8 (0.05)	2.7 (0.06)	<0.001
Fruit juice	0.3 (0.06)	0.5 (0.05)	0.7 (0.05)	0.9 (0.05)	1.0 (0.06)	<0.001
Vegetables	2.9 (0.10)	3.3 (0.10)	3.6 (0.09)	4.1 (0.10)	5.7 (0.11)	<0.001
Whole grains	0.7 (0.06)	1.2 (0.05)	1.7 (0.05)	2.0 (0.05)	2.7 (0.06)	<0.001
RG w/o sweets^a^	5.5 (0.08)	4.2 (0.08)	3.7 (0.08)	3.3 (0.08)	2.8 (0.08)	<0.001
RG sweets^b^	0.9 (0.04)	0.8 (0.04)	0.7 (0.04)	0.7 (0.04)	0.6 (0.04)	<0.001
Red meat	3.0 (0.09)	3.0 (0.08)	2.7 (0.08)	2.5 (0.08)	1.9 (0.09)	<0.001
Processed meat	1.4 (0.04)	1.4 (0.04)	1.2 (0.04)	1.1 (0.04)	0.9 (0.04)	<0.001
Fish and seafood	0.6 (0.06)	0.9 (0.06)	1.0 (0.05)	1.3 (0.06)	1.5 (0.06)	<0.001
Poultry	0.9 (0.06)	1.2 (0.06)	1.2 (0.06)	1.3 (0.06)	1.4 (0.06)	<0.001
Eggs	0.6 (0.03)	0.6 (0.03)	0.7 (0.03)	0.6 (0.03)	0.6 (0.03)	0.09
Legumes	0.1 (0.01)	0.2 (0.01)	0.2 (0.01)	0.3 (0.01)	0.3 (0.01)	<0.001
Nuts/seeds	0.4 (0.05)	0.5 (0.05)	0.7 (0.05)	0.9 (0.05)	1.2 (0.05)	<0.001
SSBs	2.2 (0.07)	1.7 (0.06)	1.2 (0.06)	1.1 (0.06)	0.8 (0.07)	<0.001
Diet beverages	0.3 (0.05)	0.4 (0.05)	0.5 (0.05)	0.5 (0.05)	0.4 (0.05)	0.09
Candy, sugars^c^	2.3 (0.09)	2.0 (0.09)	1.9 (0.09)	1.7 (0.09)	1.6 (0.09)	<0.001
Coffee, tea	2.0 (0.19)	2.0 (0.18)	2.0 (0.18)	1.5 (0.18)	1.7 (0.19)	0.09

Abbreviations: CHO, carbohydrates; MUFA, monounsaturated fatty acids; PUFA, polyunsaturated fatty acids; RG, refined grain; SFA, saturated fatty acids; SSBs, sugar sweetened beverages.

* Adjusted for age, sex, race, field centre, education and energy intake.

^a^ Refined grain w/o sweets include white breads, rolls, buns, flour tortillas, crackers, pasta and white race.

^b^ Refined grain sweets include cake, cookies, pie, donuts and pastries.

^c^ Candy, sugars include any candy, sugar, honey, syrup, jams/jelly/preserves and other sweet condiments.

In sensitivity analysis, we compared baseline characteristics between participants who attended Y25 clinic exam and those who did not and between those who had a CT scan and those who did not. Although Y25 attendees were 1 year older than non‐attendees, there was no significant difference in HEI2015 diet quality, physical activity, or BMI at baseline. Similar results were also observed for those who had a CT scan and those who did not. In another sensitivity analysis, the Y25 sample (*n* = 3017) for anthropometric measures weight, BMI, and waist circumference and change in these measures was enriched with Y20 data for participants who did not attend the Y25 clinic exam (*n* = 454) for total *n* = 3471. Finally, we used generalized additive models (SAS Proc GAM) to assess if associations between diet quality and body composition measures were nonlinear.

## Results

Baseline characteristics stratified across quintiles of baseline HEI2015 are shown in Table 1. Compared with participants in the lowest HEI2015 quintile, those in the highest quintile were more likely to be women and White, were older, reported more years of education and more physical activity, and fewer reported current smoking. Baseline BMI, weight, and waist circumference were similar across quintiles of HEI2015. Unadjusted means (SD) for HEI2015 diet quality and physical activity scores for each of Y0, Y7 and Y20 are shown in Table S1. Over the years, diet quality improved while physical activity declined.

Baseline dietary intakes including nutrients and food groups stratified across quintiles of HEI2015 are shown in Table 2. Intakes of polyunsaturated fatty acids, n3 fatty acids, protein and fibre were higher, while intakes of energy, saturated fatty acids and added sugar were lower among those in the highest HEI2015 quintile compared with the lowest quintile. Food group intakes follow the HEI2015 scoring.

As shown in Table 3, in the fully adjusted models, averaged (Y0, Y7 and Y20) HEI2015 was significantly and inversely associated with weight, BMI, and waist circumference, as well as weight gain, change in BMI, and increase in waist circumference over the 25‐year follow‐up. After enriching the sample of Y25 data (*n* = 3017) with Y20 data for participants who did not attend the Y25 clinic exam (*n* = 454), all associations between HEI2015 and anthropometric measures were strengthened (*P*_trend_ ≤ 0.001) (Table S2). Similarly, HEI2015 was inversely associated with Y25 VAT (*P*_trend_ < 0.001). However, we observed a null association between HEI2015 and the VAT/SAT ratio. Total muscle mass volume declined slightly across HEI2015 quintiles (*P*_trend_ = 0.03), however, lean muscle volume was similar across quintiles (*P*_trend_ = 0.55). IMAT and IMAT/total muscle mass volume ratio declined with better diet quality, which explains the lower total muscle volume in those with better diet quality. Furthermore, muscle quality (less lipid within the muscle) increased across quintiles of HEI2015. A significant nonlinear but inverse association between HEI2015 and SAT (*P* = 0.011) was observed (Figure S1). All HEI2015‐body composition associations were attenuated when adjusted for mediators hormone replacement therapy use and prevalent hypertension, high cholesterol, diabetes and cardiovascular disease (data not shown).

**Table 3:** Anthropometric measures and CT‐measured muscle mass and adipose tissue stratified across averaged HEI2015 diet quality score among adults enrolled in CARDIA, *n* = 3017

	Quintiles of averaged (Year 0, 7, and 20) HEI2015 diet quality score					
Body composition measures at Y25	1 (*n* = 603)	2 (*n* = 604)	3 (*n* = 603)	4 (*n* = 604)	5 (*n* = 603)	
HEI2015 score (range)	44.1 (28.0 < 49.2)	52.3 (49.2 < 55.3)	58.2 (55.3 < 60.8)	63.8 (60.8 < 67.2)	72.7 (>67.2)	*P*_trend_
*Anthropometric measures by Year25* *						
Weight, lb at y25	191.3 (1.85)	194.7 (1.75)	197.0 (1.72)	192.2 (1.74)	186.4 (1.88)	0.05
BMI, kg/m^2^	29.9 (0.29)	30.5 (0.28)	30.8 (0.27)	30.1 (0.27)	29.1 (0.30)	0.05
Waist, cm	94.7 (0.63)	95.3 (0.60)	94.7 (0.59)	94.1 (0.60)	91.3 (0.64)	<0.001
*25‐year change in anthropometric measures* **						
Weight gain by Y25, lb	37.3 (1.37)	38.5 (0.30)	38.1 (1.28)	34.9 (1.29)	32.9 (1.39)	0.01
Change in BMI, kg/m^2^	5.8 (0.22)	6.1 (0.21)	6.0 (0.20)	5.4 (0.20)	5.0 (0.22)	0.005
Change in waist, cm	17.5 (0.49)	17.6 (0.46)	17.3 (0.45)	16.3 (0.46)	15.2 (0.49)	<0.001
*CT measures of muscle composition and abdominal adipose tissue* *						
*Muscle composition* *						
Total muscle vol, cm^3^	20.46 (0.15)	20.57 (0.14)	20.68 (0.14)	20.40 (0.14)	19.99 (0.15)	0.03
Lean muscle vol, cm^3^	17.98 (0.12)	18.10 (0.11)	18.19 (0.11)	18.07 (0.11)	17.97 (0.12)	0.55
IMAT volume, cm^3^	9.52 (0.25)	9.47 (0.24)	9.53 (0.24)	8.92 (0.24)	8.12 (0.26)	<0.001
IMAT/Total muscle ratio	0.136 (0.003)	0.135 (0.003)	0.136 (0.003)	0.128 (0.003)	0.116 (0.003)	<0.001
IMAT/Lean ratio	0.114 (0.002)	0.114 (0.002)	0.114 (0.002)	0.109 (0.002)	0.100 (0.002)	<0.001
Muscle quality, HU	41.1 (0.24)	40.8 (0.23)	41.0 (0.22)	41.1 (0.22)	42.2 (0.24)	0.002
*Adipose tissue* *						
VAT volume, cm^3^	136.8 (3.02)	136.7 (2.86)	136.3 (2.80)	128.3 (2.84)	116.6 (3.06)	<0.001
VAT/SAT ratio	0.482 (0.011)	0.455 (0.011)	0.451 (0.011)	0.450 (0.011)	0.449 (0.012)	0.08

Abbreviations: BMI, body mass index; CT, computed tomography; IMAT, intermuscular adipose tissue; VAT, visceral adipose tissue; waist, waist circumference. Muscle quality = higher score means less lipid within the muscle.

* Adjusted for age, sex, race, field centre, education, height, averaged energy intake, current smoking status, current drinking status and averaged physical activity.

^**^ Adjusted for age, sex, race, field centre, education, height, averaged energy intake, current smoking status, current drinking status, averaged physical activity, and baseline weight, BMI, or waist circumference, as appropriate.

## Discussion

In this study of over 3000 middle‐aged men and women, our findings showed better diet quality (i.e., higher HEI2015 score) associated with greater muscle quality and lower adipose tissue volumes, including VAT, SAT and IMAT, but not lean muscle volume. Furthermore, consuming a higher quality diet was also associated with better anthropometric profiles, including lower BMI, weight, and waist circumference, less weight gain, and less increase in waist circumference over 25 years of follow‐up. At baseline, participants with higher HEI2015 were more likely to be women, White, have higher education and be more physically active than those with lower HEI2015 scores.

Better body composition among adults consuming a healthy diet pattern has been reported in several studies. Consistent with our study findings, adults enrolled in the Multiethnic Cohort Study who consumed a healthy diet pattern or who improved their diet quality gained less weight over 10 years of follow‐up.^22^ In the same study, lower DXA‐measured per cent body fat, VAT and SAT were observed among those reporting higher HEI2010 scores compared with lower scores.^12^ In addition to less weight gain and less increase in waist circumference in CARDIA study participants, we also observed lower VAT, SAT and IMAT in adults reporting higher HEI2015 compared with those reporting a lower quality diet. Similarly, a modified Mediterranean‐type diet pattern was inversely associated with VAT and pericardial fat, but not SAT, in adults enrolled in the Multi‐Ethnic Study of Atherosclerosis (MESA).^13^ Australian men and women who consumed a traditional diet high in vegetables, whole grain cereals, and animal protein had high DXA‐ derived skeletal muscle index.^9^, ^10^ By comparison, men and women in CARDIA who reported a high HEI2015 score showed denser skeletal muscle (less lipid within the muscle), but lean muscle mass volume was similar across quintiles of diet quality scores.

The nutrient density of foods making up a healthy diet pattern or diet quality score may explain the beneficial associations of body composition measures.^23^ Lower intakes of energy, saturated fat, and added sugar and higher intakes of protein, n3 fatty acids, and fibre were observed among CARDIA participants consuming a high quality diet than lower quality. Dietary protein intake is considered the primary nutrient promoting and preserving muscle mass^7^, ^8^, ^24^, ^25^ while protein intake also enhances fat loss.^7^, ^26^ Among adults enrolled in the Framingham study, protein intake predicted higher appendicular lean mass, independent of the remaining dietary intake.^27^ In our study, protein intake increased across HEI2015 quintiles; however, lean muscle volume was similar across the HEI2015 quintiles suggesting sufficient protein intake in all HEI2015 quintiles. Other protein sources included in a healthy diet, such as nuts and fish, have thermogenic effects that reduce fat accumulation.^7^, ^28^, ^29^, ^30^ n3 fatty acids are also involved in muscle synthesis.^28^

In addition to protein intake, consumption of whole grains compared with refined grain products enhance net protein balance in adults.^31^, ^32^ Antioxidants found in whole grains as well as in fruit and vegetables have been considered dietary mediators that may affect skeletal muscle through depressing the catabolic effect of oxidative stress on skeletal muscle.^33^, ^34^ In middle‐aged to older US adults, higher intake of fibre was associated with greater grip strength and muscle mass, and lower BMI than among those who consumed less fibre.^34^ In addition, higher whole grain intake was associated with lower VAT and SAT in middle‐aged adults.^35^ Though experimental human studies of added sugar and muscle mass have not been conducted, a feeding study in mice showed that excess added sugar induced attenuated muscle mass.^36^ Furthermore, added sugar and sugar‐rich foods and beverages were associated with weight gain and greater BMI, waist circumference, and adipose tissue volumes.^37^, ^38^

Our study has strengths and limitations. First, dietary intake was self‐reported; however, trained and certified diet interviewers administered a validated diet history questionnaire three times over 20 years.^15^, ^39^ And compared with a food frequency questionnaire, for example, the Diet History collects more detailed information, including brand name information and specific foods and beverages consumed. Typically, energy dense snack foods are under‐reported by most adults, including lean, overweight, and obese adults^40^; therefore, the strength of the associations between diet quality and body composition measures would be attenuated. The HEI2015, reflecting diet quality, has been validated.^16^ Although we have only one CT scan of the abdominal region, this provides a precise image of the regional adipose and muscle mass tissue volumes.^18^, ^19^ Despite these limitations, this study has many strengths. First, the CARDIA study is prospective in design with over 3000 Black and White men and women participating in numerous clinic exams over 25 years of follow‐up. Dietary intake was assessed three times, including baseline, Y7 and Y20, which would take into account the changing food supply. Anthropometrics were measured by trained and certified data collectors at each clinic exam which allowed reporting of change in weight and waist circumference over time. Finally, CT scans provide precise images of adipose tissue depots and muscle mass. Trained and certified staff used state‐of‐the‐art software to quantify adipose tissue depots and muscle mass.

Generalization of the findings may be limited to middle‐aged Black and White adult men and women. Although we included covariates that may be mediators in the pathway between diet quality and body composition, there may be other factors that were not captured in this study. Moreover, this study was conducted among both men and women providing the opportunity to test the modifying role of sex between diet and body composition, although sex did not modify the diet‐body composition associations (*P*_interaction_ > 0.30).

In conclusion, our study findings suggest that higher HEI2015 is associated with less weight gain and less increase in waist circumference, lower VAT, SAT, and IMAT volumes and better muscle quality in middle‐aged adults. However, lean muscle mass was similar across quintiles of HEI2015 diet quality score. Improving diet quality in young to middle‐aged adults is a recommended strategy to promote better measures of body composition. Our study findings support the 2020–2025 DGAs and suggest that healthier food choices may influence body composition.^41^

## Funding

The Coronary Artery Risk Development in Young Adults Study (CARDIA) is conducted and supported by the National Heart, Lung, and Blood Institute (NHLBI) in collaboration with the University of Alabama at Birmingham (HHSN268201800005I and HHSN268201800007I), Northwestern University (HHSN268201800003I), University of Minnesota (HHSN268201800006I), and Kaiser Foundation Research Institute (HHSN268201800004I). This work was also supported by R21 HL135300 (LM Steffen) and R01 HL150053 (LM Steffen). This manuscript has been reviewed by CARDIA for scientific content.

## Conflict of interest

All authors confirm that they do not have any conflicts of interest to disclose.

## Supporting information


**Table S1.** Unadjusted HEI2015 scores and physical activity score at exam years 0, 7, and 20 stratified across quintiles of averaged HEI2015 score, *n*=3,017.
**Table S2.** Association between HEI2015 diet quality and anthropometric measurements at Year 20 and Year 25 clinic exams, n=3,471.
**Figure S1.** Nonlinear association between HEI2015 diet quality score and subcutaneous adipose tissue volume (cm^3^) in CARDIA participants, *n*=3017.

## Data Availability

The data and materials supporting the conclusions of this article are available by contacting the CARDIA Coordinating Center (website https://www.cardia.dopm.uab.edu/).
