# Supplementary material for: Diet quality is associated with adipose tissue and muscle mass: the Coronary Artery Risk Development in Young Adults (CARDIA) study
Source: J Cachexia Sarcopenia Muscle. 2023 Dec 12;15(1):425–33. doi: 10.1002/jcsm.13399 (PMC10834311; doi:10.1002/jcsm.13399)
Supplement: Supplementary file 1 — Table S1. Unadjusted HEI2015 scores and physical activity score at exam years 0, 7, and 20 stratified across quintiles of averaged HEI2015 score, n=3,017. Table S2. Association between HEI2015 diet quality and anthropometric measurements at Year 20 and Year 25 clinic exams, n=3,471. Figure S1. Nonlinear association between HEI2015 diet quality score and subcutaneous adipose tissue volume (cm3) in CARDIA participants, n=3017. [file JCSM-15-425-s001.docx]

Table S1. Unadjusted HEI2015 scores and physical activity score at exam years 0, 7, and 20 stratified across quintiles of averaged HEI2015 score, n=3,017

|  | Quintiles of averaged HEI2015 diet score | | | | |  |
| --- | --- | --- | --- | --- | --- | --- |
| Quintile | 1  (n=603) | 2  (n=604) | 3  (n=603) | 4  (n=604) | 5  (n=603) | p_trend_ |
| Averaged HEI2015 | 44.1  (28.0<49.2) | 52.3  (49.2<55.3) | 58.2  (55.3<60.8) | 63.8  (60.8<67.2) | 72.7  (>67.2) |  |
| HEI2015 diet score | | | | | |  |
| Y0 | 43.0(6.69) | 49.9(6.48) | 54.8 (6.72) | 59.9 (7.49) | 69.4 (8.08) | <0.001 |
| Y7 | 43.1 (7.25) | 51.4 (6.86) | 56.6 (6.82) | 62.9 (7.52) | 71.6 (7.69) | <0.001 |
| Y20 | 47.1 (7.96) | 56.4 (7.73) | 64.0 (7.44) | 69.3 (7.68) | 77.5 (7.48) | <0.001 |
| Physical activity score | |  |  |  |  |  |
| Y0 | 369.3(293.5) | 390.0(297.3) | 399.5(281.5) | 441.8(301.5) | 483.5(297.9) | <0.001 |
| Y7 | 284.5(256.4) | 320.7(259.2) | 315.6(248.4) | 356.5(269.0) | 415.9(295.7) | <0.001 |
| Y20 | 265.6(246.6) | 308.8(255.8) | 322.3(265.8) | 360.9(283.3) | 419.1(288.2) | <0.001 |

Table S2. Association between HEI2015 diet quality and anthropometric measurements at Year 20 and Year 25 clinic exams, n=3,471

| Y20 and Y25  Anthropometrics | Quintiles of averaged HEI2015 diet quality score | | | | | | | | |  |
| --- | --- | --- | --- | --- | --- | --- | --- | --- | --- | --- |
|  | 1 | | 2 | | 3 | | 4 | | 5 |  |
| HEI2015 score (SE),  Range | 42.5(4.16)  <47.7 | | 50.9(1.74)  47.8,53.8 | | 56.9(1.69)  53.9,59.8 | | 62.9(1.89)  59.9,66.3 | | 72.4(4.53)  >66.3 | p_trend_ |
| *Anthropometric Measures (Y20 + Y25)** | | | | | | | | | |  |
| total n=3,471 | (n=694) | (n=694) | | (n=695) | | (n=694) | | (n=694) | |  |
| Weight, lb | 193.4(1.77) | 196.8(1.68) | | 198.2(1.65) | | 191.3(1.67) | | 183.4(1.78) | | <0.001 |
| BMI, kg/m^2^ | 30.3 (0.28) | 30.8 (0.27) | | 31.1 (0.26) | | 29.9 (0.26) | | 28.7 (0.28) | | <0.001 |
| Waist, cm | 95.5 (0.61) | 96.1 (0.58) | | 95.9 (0.57) | | 93.8 (0.58) | | 90.1 (0.61) | | <0.001 |
| *20-25-year Change in Anthropometric Measure* | | | | | | | | | |  |
| Weight gain, lb | 38.3 (1.29) | | 39.3 (1.22) | | 38.2 (1.20) | | 34.5 (1.22) | | 31.1 (1.30) | <0.001 |
| Change in BMI, kg/m^2^ | 6.0 (0.20) | | 6.2 (0.19) | | 6.0 (0.19) | | 5.4 (0.19) | | 4.8 (0.21) | <.0001 |
| Change in waist, cm | 18.1 (0.51) | | 18.2 (0.46) | | 17.3 (0.42) | | 16.4 (0.42) | | 14.5 (0.44) | <0.001 |

Adjusted for age, sex, race, field center, education, height, averaged energy intake, current smoking status, current drinking status, and averaged physical activity

BMI, body mass index

*Y20 n=454; Y25 n=3,017

Figure S1. Nonlinear association between HEI2015 diet quality score and subcutaneous adipose tissue volume (cm^3^) in CARDIA participants, n=3017


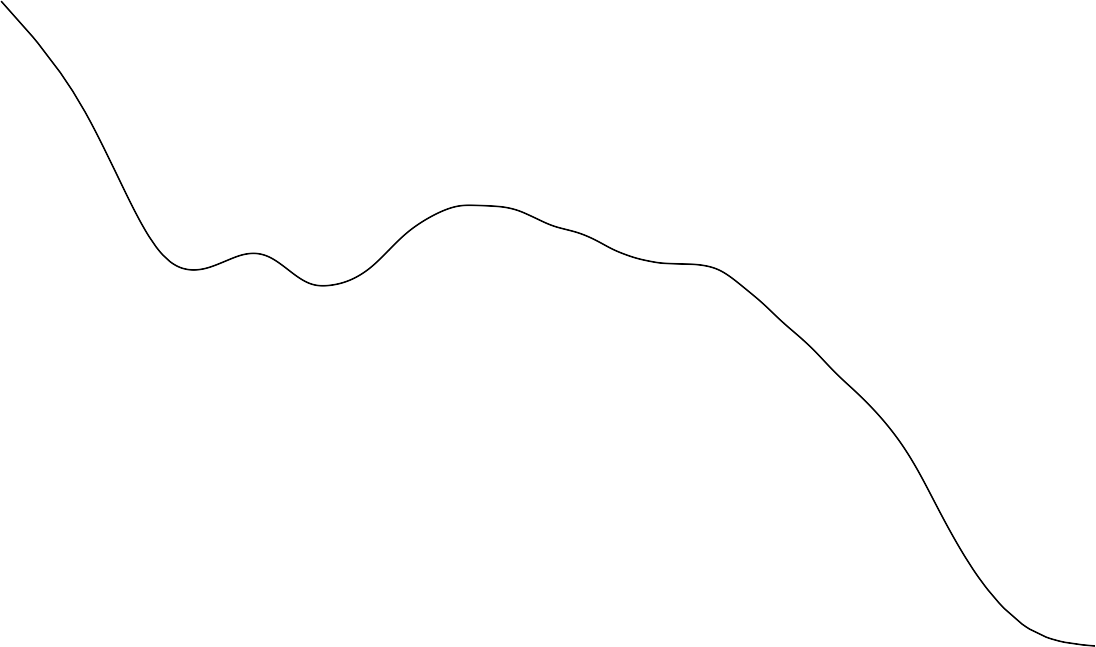


50

Linearity p = 0.005

Nonlinearity p=0.011

25

0

-25

-50

-75

40

60

Average HEI2015

80

f (average HEI2015)
